# Land Use, Yield and Quality Changes of Minor Field Crops: Is There Superseded Potential to Be Reinvented in Northern Europe?

**DOI:** 10.1371/journal.pone.0166403

**Published:** 2016-11-21

**Authors:** Pirjo Peltonen-Sainio, Lauri Jauhiainen, Heikki Lehtonen

**Affiliations:** 1 Natural Resources Institute Finland (Luke), Management and Production of Renewable Resources, FI-31600 Jokioinen, Finland; 2 Natural Resources Institute Finland (Luke), Economics and Society, FI-00790 Helsinki, Finland; University of Delhi, INDIA

## Abstract

Diversification of agriculture was one of the strengthened aims of the greening payment of European Agricultural Policy (CAP) as diversification provides numerous ecosystems services compared to cereal-intensive crop rotations. This study focuses on current minor crops in Finland that have potential for expanded production and considers changes in their cropping areas, yield trends, breeding gains, roles in crop rotations and potential for improving resilience. Long-term datasets of Natural Resources Institute Finland and farmers’ land use data from the Agency of Rural Affairs were used to analyze the above-mentioned trends and changes. The role of minor crops in rotations declined when early and late CAP periods were compared and that of cereal monocultures strengthened. Genetic yield potentials of minor crops have increased as also genetic improvements in quality traits, although some typical trade-offs with improved yields have also appeared. However, the gap between potential and attained yields has expanded, depending on the minor crop, as national yield trends have either stagnated or declined. When comparing genetic improvements of minor crops to those of the emerging major crop, spring wheat, breeding achievements in minor crops were lower. It was evident that the current agricultural policies in the prevailing market and the price environment have not encouraged cultivation of minor crops but further strengthened the role of cereal monocultures. We suggest optimization of agricultural land use, which is a core element of sustainable intensification, as a future means to couple long-term environmental sustainability with better success in economic profitability and social acceptability. This calls for development of effective policy instruments to support farmer’s diversification actions.

## Introduction

In Finland, spring cereals, barley (*Hordeum vulgare* L.), oat (*Avena sativa* L.) and also increasingly wheat (*Triticum aestivum* L.) dominate land use. Cereal monocultures are typical in the southern prime production regions of the country with many recorded adverse effects on crop yields and the environment [1]. On the other hand, due to the striking south-north division of crop and dairy production, grasslands are common in the landscapes of the central and northern parts of Finland. Spring cereals and grass crops together account for 80% of cultivated area, thereby leaving a limited land area of only ~120.000 hectares for the following minor crops (S1 Fig): turnip rape (*Brassica rapa* L.) and oilseed rape (*B*. *napus* L.), together referred to as rapeseed, potato (*Solanum tuberosum* L.), pea (*Pisum sativum* L.), winter rye (*Secale cereale* L.), winter wheat, sugar beet (*Beta vulgaris* var. *altissima*) and also recently faba bean (*Vicia faba* L.).

**Fig 1:**
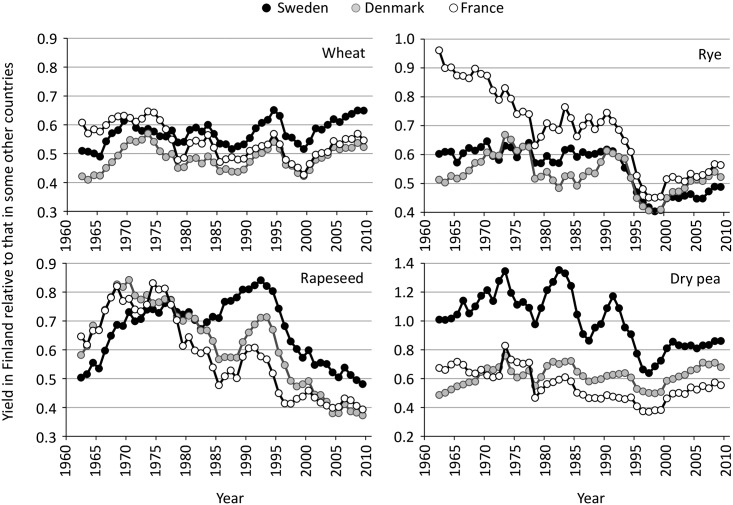
Yields of winter rye, rapeseed and field pea in Finland relative to those in Sweden, Denmark and France during 1961–2012 (five-year moving averages) with wheat (both spring and winter) as a reference of major crop. Data from FAO [3].

Yield difference of both major and minor crops between Finland and countries like Sweden, Denmark and France is apparent owing to the high latitude conditions coupled with exceptionally short growing season. Possible shifts in yield differences over time may reveal differences in regional capacities and economic incentives to develop crops and cropping systems. These are significantly affected by markets, prices and agricultural policies as well as public research and development (R&D) funding directed to development of minor crops. Contrary to the relatively constant yield difference since the 1960s in wheat and the recently reduced yield difference in barley between Finland and other countries [1], yield gap has dramatically expanded for rapeseed, especially since the 1990s (Fig 1) [2]. The situation is the same with winter rye and pea, though in general, the yield difference between Finland and other countries in pea yields are slightly lower than that for winter rye and particularly rapeseed. All these findings and the reduction in area under minor crops emphasize their potentially reduced competitiveness in Finland.

Diversification of agricultural production was one of the strengthened aims of the greening payment of European Common Agricultural Policy (CAP), Pillar 1 [4]. Diversified land use is considered to provide many valuable ecosystem services for European agriculture—and is also a response to global concern about biodiversity loss [5]. Switching from cereal-intensive crop rotations towards diverse cropping sequencing benefits the environment, e.g. through better nutrient management, reduced need for nitrogen (N) fertilizers, increased biodiversity, improved soil conditions and functions and reduced pest, disease and weed risks [6–8]. Especially N fixing legumes are appreciated as break-crops [9–11]. Diversification is also one of the core means to increase resilience of crop production to weather variability [12]. To capitalize on the benefits from diversification and to better design crop rotations, eco-efficiency (the ratio between environmental impact and economic value) needs to be assessed [8]. Diversified land use is a key element of sustainable intensification of cropping systems, that need to be designed locally [13], to reduce yield gaps and gain environmental sustainability, economic profitability and social acceptability [1].

Owing to the negligible cultivation area and limited markets of minor crops, plant breeding efforts have been directed towards major crops, while abandoning minor crops [14]. Such a process focusing on a smaller number of economically important primary crops has been further promoted for commercial reasons, as governmental breeding companies have been privatised and also merged [14]. This may have created a bottleneck where the current low cultivation area discourages investment in breeding programs of minor crops, while the lack of competitive cultivars discourages farmers to diversify their cropping systems and expand cultivation of minor crops.

To only consider the role of breeding in sustaining or superseding minor crop production is evidently too one-sided. The limited demand of minor crops at local markets, the specific management and marketing skills needed, higher labour input and production costs per produced unit, as well as the lower or more uncertain profitability compared to the main crops, inhibit introduction of minor crops in rotations [15,16]. This empirical observation has been confirmed in economic modelling studies as well [17,18]. Furthermore, both public and private investments have been largely allocated to the low number of major global crops, with a good repayment ratio [19], while minor crops do not success well in competition for resources for R&D [20]. One particular, emerging challenge—also experienced by Finnish farmers—is the lower number of pesticides, if any, registered and thereby, available for controlling noxious diseases, pests and weeds in minor crops [21]. Small markets and low demand for such pesticides apparently discourages suppliers. On the other hand, farm area payments decoupled from production and occasionally low market crop prices compared to the production costs discourage farmers from using pesticides [18]. This is critical for the existence of minor crops in crop rotations, as biotic stressors are among the major factors causing yield instability and even total crop failures for minor crops [22].

All the fundamental flaws associated with understating of minor crops pose a challenge to find the means to expand their cultivation and to increase their attractiveness for companies investing in the developing processes in one way or another. In the case of Finland, minor crops, e.g. pea, faba bean and rapeseed as a protein source can be increased in feed use and be a substitute for imported soybean meal [*Glycine max* (L.) Merr.]. The main concern, however is the high production cost compared to imported protein feed. Hence, governmental organizations and agricultural policies have an important role in developing feasible incentives and means for farmers to expand cultivation of minor crops. One encouraging example is that the National Emergency Supply Agency in Finland supports breeding programs of minor crops until a crop becomes economically profitable for the national breeding company.

With this study we aimed to: 1) assess the genetic yield and quality improvements achieved in crops having a permanent but minor role in Finnish agriculture and to evaluate the gaps between minor crop potential and actual farm-yields when compared to those of major crops, 2) to identify possible changes in the role of minor crops in crop rotations in the case study region that is characterized as the area with the most potential for cultivation of special crops in Finland, and 3) to characterize the differences in responsiveness of yield of a number of minor and major crops to variable growing conditions and thereby, to characterize the general potential to improve resilience of northern European cropping systems to weather variability through diversified land use with minor crops.

## Materials and Methods

### Assessment of yield and quality trends

#### Study period

The study period was divided into various sub-periods as described by Peltonen-Sainio et al. [1] as agriculture has undergone periodic changes with likely impacts on yield trends. In the first period of 1970–1980, agriculture was strongly mechanized and pre-modern agricultural practices were developed and largely implemented. For example, during this period the number of tractors per unit land area more than doubled and the number of combine harvesters increased six-fold [3]. Also, use of industrial fertilizers increased from ca. 20 kg N ha^-1^ and 40 kg P ha^-1^ to 80 kg of both N and P ha^-1^ and the method of fertilizer placement was implemented. The second period of 1981–1994 was characterized by introduction of modern crop management practices, chemical control of pests and diseases, introduction of plant growth regulators, etc., which all intensified production. However, use of agro-chemicals has been modest in Finland due to lower pest and disease outbreak pressure and because the market and policy incentives have not favoured their use. The latest period of 1995–2013 was characterized as the period of Finland being a member of European Union (EU) implementing CAP, launching the AEP (Agri-Environment Program), structural aids and thereby, creating large changes in the socio-economic environment for practising agriculture.

#### Experimental arrangements and measurements

Data on national mean yields and production areas, provided by Statistical Services of the Natural Resources Institute Finland (Luke), have been available since 1920 depending on introduction of a crop into cultivation in Finland at a noteworthy scale (S1 Fig). In addition, data on multi-location Luke Official Variety Trials for winter wheat and rye, turnip rape, oilseed rape, field pea and timothy (*Phleum pratense* L.) were available for 1970–2013 and for potato for 1970–2004. The total number of locations was 30, but the sites depended on year and crop. Each crop was grown in its most typical region. The experiments, measurements and analyses followed the procedures specified in Laine et al. [23].

All experiments were arranged as randomized complete block designs or incomplete block designs. The numbers of replicates were three to four, depending on location and year. Each year the tested set of cultivars and breeding lines changed, but long-term check cultivars were used. Annual turnover of cultivars and breeding lines was typically 10–40%. The selection of lines and cultivars also differed within any one year, depending on location. Only entries that were early enough to mature at each location were included in experiments. In addition to this, to avoid biased interpretation of results, experiments producing less than 750 kg seed yield ha^-1^ were excluded as they often resulted from some errors in organising and managing the crop stands. In total, the multi-location data included 228 winter wheat, 222 winter rye, 311 turnip rape, 259 oilseed rape, 262 field pea, 431 potato and 191 timothy cultivars and lines and the total numbers of cases (n) were 3416, 5574, 4245, 2388, 3541, 3193 and 6590, respectively.

Plots were 7–10 m × 1.25 m, depending on location and year. Seeding rate ranged from 450–500 viable seeds m^-2^ for winter cereals except being 200–250 for hybrid rye, 200–350 for rapeseed, 100–140 for pea and 3000 for timothy, conforming to the commonly used sowing rates in Finland. The number of seed potatoes planted was 4–7 m^-2^. Weeds and pests were chemically controlled with the common commercial agents used at each time period. Fertilizer use depended on crop, cropping history, soil type and fertility. Fertilizer N application rate did not change over time for pea (49 kg ha^-1^), but rose slightly for rapeseed (by 6 kg N ha^-1^) and more so for winter wheat (by 19 kg N ha^-1^) and winter rye (by 23 kg N ha^-1^) due to advances in yield potentials and quality requirements. For timothy the N fertilizer rate was 185 kg N ha^-1^ but increased to 220 kg N ha^-1^ since 2000, while for potato the N rate remained constant at 70 kg N ha^-1^. For all crops phosphorus fertilizer application rates declined to correspond with the reductions in national P use.

Grain and seed yields were harvested with a combine harvester and weighed (kg ha^-1^) after removing straw, weed seeds and other particles. Grain moisture content (%) was determined by weighing the grain samples before and after oven drying, or more recently using a Dickey John apparatus. For cereals and pea the yield was adjusted to 15% grain or seed moisture content, while for rapeseed to 9% seed moisture content and for timothy as dry matter yields [23]. Potato yield was measured as total fresh yield (kg ha^-1^). Single grain or seed weight (mg) for winter cereals, rapeseed and pea was measured from samples of 100 grains/seeds each five times, disregarding the lowest and highest values from the mean. Seed protein concentration (%) was analysed for the same crops by using the Kjeldahl-method. Seed oil content (%) was determined for rapeseed with heptane–alcohol extraction and converted to dry matter. Share of commercially acceptable yield (%) was measured for potato to include only healthy, mechanically or otherwise undamaged tubers of 35–70 mm in size. Also, sensitivity of potato to discoloration of raw (10 tubers per sample) and boiled tubers (25 tubers) was measured by halving each tuber and visually scoring the colour from one to nine.

#### Statistical analyses

The statistical analysis was used to show two types of yield trends according to Luke Official Variety Trials: genetic yield improvements representing the attainable potential yields and harvested yields (i.e., realised yield potentials). A mixed model technique was used for this purpose using the following statistical model (1):

$$y_{ijkl}=\mu +\alpha_{j}+\beta_{k}+\gamma_{jkl}+\eta_{i}+\varepsilon_{ijkl}$$ (1)

where y_ijkl_ is the observed seed yield of the i^th^ cultivar cultivated in the j^th^ location, the k^th^ year (k = 1970,…, 2013) and the l^th^ trial, μ is the intercept, α_j_ is the random effect of the j^th^ experimental site, β_k_ is the fixed effect of the k^th^ year, γ_jkl_ is the random effect of the jkl^th^ trial, η_i_ is the fixed effect of the i^th^ cultivar and ε_ijkl_ is the residual error. The assumptions for the random effects were: $\alpha_{j}\sim iid\;N\left(0,\delta_{location}^{2}\right)$, $\gamma_{jkl}\sim iid\;N\left(0,\delta_{trial}^{2}\right)$, *ε*_*ijkl*_~*iid N*(0,*δ*^2^) and all the effects are independent of each other. Annual breeding improvements were calculated comparing the estimated seed yields of different cultivars, ${\hat{\eta }}_{i}$ to the year in which each cultivar was entered in the trials. The parameters of the models were estimated using the restricted maximum likelihood (REML) method with the SAS system and MIXED procedure [24]. The same statistical model was used to analyse trends in all the quality traits measured. Scatter plots of residual and fitted values revealed some outliers, but because of their small number, they did not influence the results. Graphical examination of data also revealed some clear outlier records, which were removed before analysis.

#### Trend assessments

We applied the definitions of Fischer [25] when characterizing national mean yield as the actual yield, and potential yield as a measured yield of the best cultivars cultivated by following best practices. Changes in national grain yields (±kg ha^-1^ ya^-1^) were estimated by measuring the change from each single preceding year to the next, transforming the data to five-year moving averages and thereafter calculating the average change in national grain yield as a mean across all the years of each study period. Changes in genetic yield potential (±kg ha^-1^ ya^-1^) were estimated in a similar way, but by comparing the genetic yield improvements in yielding capacity of new cultivars to their predecessors. Cultivars were classified as new cultivars in the first year of introduction into Luke Official Variety Trials. The degree of realisation of the genetic yield potential (±% unit ya^-1^) was based on comparison of national yields from Luke Statistics Services with the genetic yield potential estimated from Luke Official Variety Trials.

### Differences among crops in their responsiveness to variable conditions

Average yield level was calculated for all crops cultivated at the same experimental site in the same year. A correlation matrix was created by calculating the correlation between each pair of crops using average yields. The correlation matrix was used in the factor analysis to find inter-correlation between yield profiles of different crops. SAS/FACTOR and CORR-procedures were used to perform this analysis.

### Changes in land use of minor and major crops

Data on land use from the Agency of Rural Affairs (Mavi) consisted of information on field crops cultivated in each of the field parcels and, when appropriate, field parcels were further divided to agricultural parcels each differing in crop species. We used data from the south-western primary production region of Finland representing an area having the highest potential for cultivation of special crops and for diversification of cropping systems. The data was divided into two five-year periods: the first period since Finland joined the EU (1995–1999) and the second period of 2007–2011. In total, this area consisted of 240 000 and 280 000 agricultural parcels in the first and second periods, respectively. For all field parcels the count of occurrence of each crop was calculated. The calculation was made separately for both periods. Distribution of counts was presented as a weighted average according to the area of the parcel.

## Results

### Trends in realized yields and genetic yield potentials

**Fig 2:**
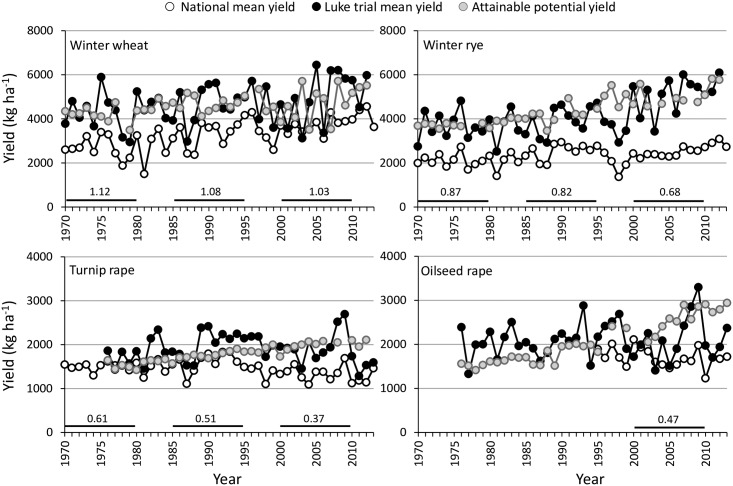
Changes in national yields, yields in Luke Official Variety Trials and in potential yields of winter wheat, winter rye, turnip rape and oilseed rape in 1970–2012. The numbers shown for different time periods (1970–1980, 1985–1995 and 2000–2010) indicate the national mean yield of each crop compared to that of spring wheat as a reference of major crop with expanded cultivation areas. National data from Luke Statistics Services [26].

**Fig 3:**
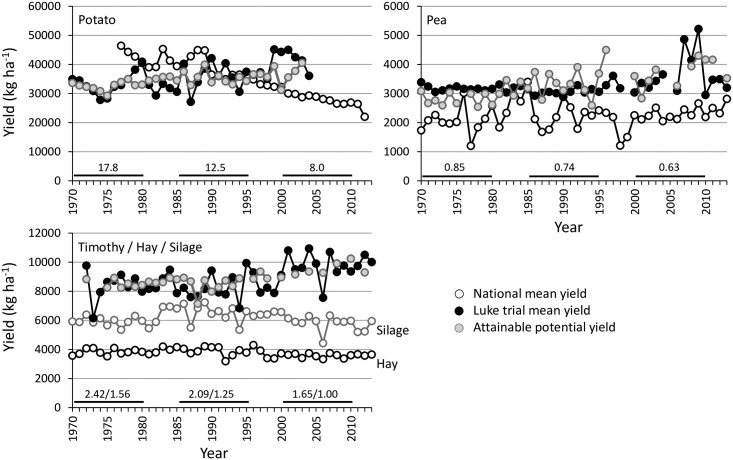
Changes in national yields, yields in Luke Official Variety Trials and in potential yields of potato, field pea and timothy when harvested for silage or hay in 1970–2012. The numbers shown for different time periods (1970–1980, 1985–1995 and 2000–2010) indicate the national mean yield of each crop compared to that of spring wheat as a reference of major crop with expanded cultivation areas. National data from Luke Statistics Services [26].

Interannual variation was large in national and experimental yields of minor grain and seed crops (Figs 2 and 3). When comparing the national yields of minor crops to that of spring wheat, it was apparent that the national yields of all minor crops have advanced less compared to that of spring wheat. National yields of winter cereals have advanced most, averaging 1.15% ya^-1^ in wheat and 0.93% ya^-1^ in rye, but even by 2.97 and 1.89% ya^-1^ during the period of 1981–1994 exhibiting the highest advances (Table 1). 1981–1994 was the only period when positive changes were recorded for turnip rape in national yields (0.86% ya^-1^). In potato, national yield declines were evident throughout the <40-year study period (Table 1, Fig 3). Increases in productivity of grasslands (0.75–0.94% ya^-1^) and pea (1.47% ya^-1^) were again at their highest during the first study period, 1961–1980. Since 1995 in all minor crops, the pace of improvements in the national yields reduced (winter wheat and rye) or yields declined (turnip rape, potato, hay and silage) compared to the preceding period, except for pea which already earlier experienced the levelling off in national yield.

**Table 1:** Changes in national mean yields, genetic yield improvements and in degree of realization of yield potentials for spring and winter cereals, spring turnip rape and oilseed rape, field pea and hay and silage. National yield improvements are given in kg and % year^-1^. Figures for the highest yield improvement for any of the sub-periods are shown in parentheses.

Period	Spring sown/planted crops				Overwintering crops			
	Turnip rape	Oilseed rape	Pea	Potato	Wheat	Rye	Hay	Silage^a^
Change in national yield (kg year^-1^)								
1970–1980	−2	·	30	−1460	−22	6	29	55
1981–1994	13	·	6	−390	93	45	−4	25
1995–2013	−19	−7	11	−631	30	12	−12	−40
Whole period (kg year^-1^)	−4	·	14	−549	37	22	1	7
Whole period (% year^-1^)	−0.27 (0.86)	−0.40 (−0.40)	0.65 (1.47)	−1.56 (−0.98)	1.15 (2.97)	0.93 (1.89)	0.03 (0.75)	0.11 (0.94)
Genetic improvement (kg year^-1^)								
							Timothy^b^	
1970–1980	4	17	11	226	−14 ^a^	9	4	
1981–1994	22	28	46	51	44	49	15	
1995–2013	14	52	28	301	40	72	32	
Whole period	17	40	31	159	31	52	22	
Change in realized yield potential (±% unit year^-1^)								
1970–1980	−1.65	·	3.79	−5.21	−0.79^a^	−0.50	6.41	0.24
1981–1994	−0.41	·	‒0.77	−1.30	1.43	0.44	−0.12	0.17
1995–2013	−1.45	−1.53	‒0.16	−2.83	0.22	−0.35	−0.40	−0.87
Whole period	−1.04	·	‒0.07	−1.96	0.45	−0.10	0.96	−0.33

^a^ Only for winter wheat, genetic yield potentials have declined though only for the period 1970–1980 and therefore, change in realized yield potential is negative

^b^ Realization of potential yield for hay and silage yields is based on comparison with genetic yield improvements for timothy (one of the prime grass crops grown in Finland), silage yield is calculated by dividing the total annual silage yield by three, the expected number of individual cuts.

For all the studied minor crops the potential yields attainable in the field conditions had an increasing trend, although some interannual variation occurred (Figs 2 and 3). Hence, genetic yield improvements were evident for all the studied time periods and all crops (Table 1). The only exception was winter wheat, for which minor genetic yield reduction averaging -14 kg ha^-1^ ya^-1^ was apparent during the first period (1970–1980). For all crops except potato, the genetic improvements in yield potential were at their lowest for the first study period. In general, genetic improvements were always higher for oilseed rape than turnip rape and for winter rye than winter wheat.

**Fig 4:**
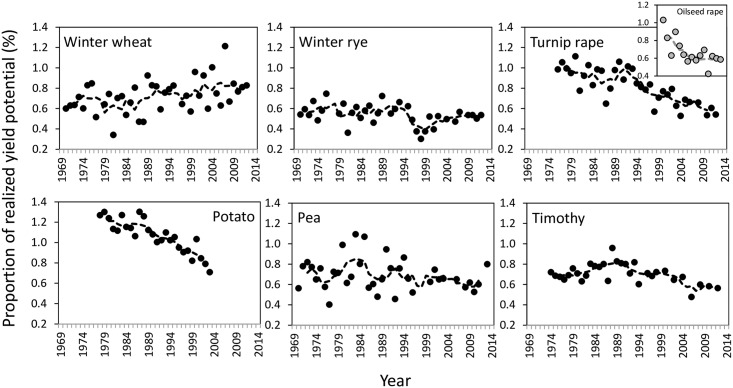
Interannual variations in realization of yield potential (i.e., a ratio between national yield and genetic yield potential) in winter cereals, rapeseed, pea and timothy (shown as black circles). Dashed line indicates five-year moving averages of the realized yield potential. National data from Luke Statistics Services [26] and genetic yield improvement from Luke Official Variety Trials.

Changes in realized genetic yield potentials were positive if the national yield advanced more compared to the change in genetic yield potential or national yield increased while genetic yield potential declined. Realized yield potential varied across years (Fig 4) mainly reflecting the fluctuations of national yields and being thereby, particularly high for winter wheat and pea (Figs 2 and 3). Throughout the study period, a clear positive trend was found only for winter wheat, while again a very dramatically declining trend was seen for turnip rape, oilseed rape and potato (Fig 4). Changes in realized yield potentials were always negative for turnip rape and potato (Table 1). For pea and hay it was negative for the two latter study periods, while for winter wheat realized yield was negative only in the first study period, while it was positive for rye only in the period of 1981–1994.

### Genetic improvements in quality traits

In general, the regional variation in acceptable quality of minor crops was high without any evidence of systematic changes (S2 Fig). Potato was the only crop showing a trend of improved quality across the years associated with modest interannual variation. For rapeseed and silage, the share of acceptable quality was high throughout the study period (data not shown). There was no evidence of marked differences in ranges of variation when means of acceptable quality in minor crops were compared to those in spring wheat, although winter cereals and pea had slightly more regional variation than other crops.

**Fig 5:**
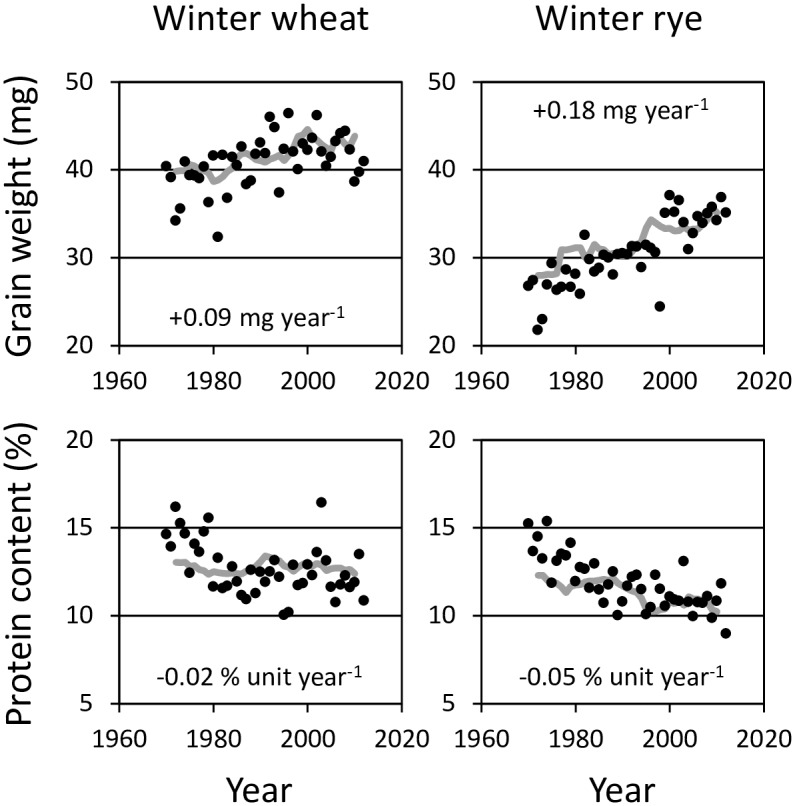
Genetic improvements in single grain weight and grain protein concentration of winter wheat and rye in 1970–2012 as five-year moving averages (grey line). Black circles indicate the interannual variations of experimental means across all the cultivars of Luke Official Variety Trials. The figure within the panel indicates the mean genetic change in quality traits.

**Fig 6:**
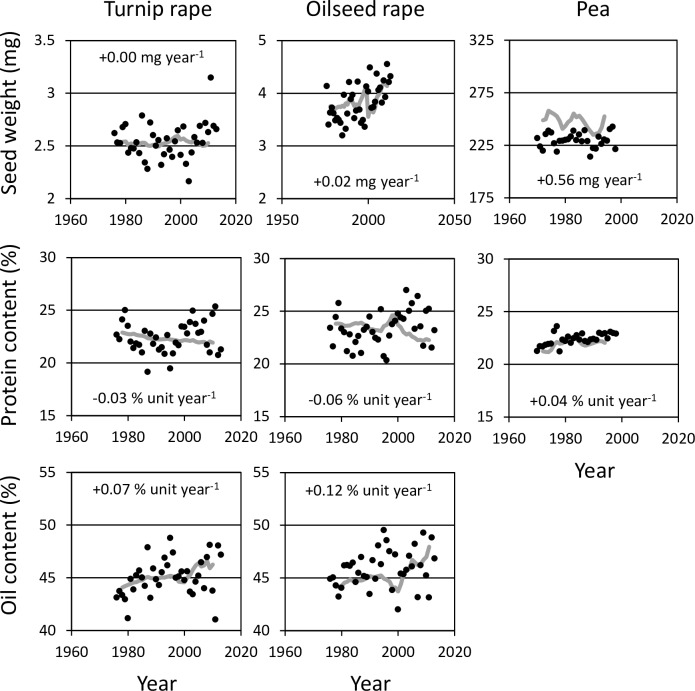
Genetic improvements in single seed weight and seed protein concentration of rapeseed and pea as well as seed oil concentration in rapeseed in 1970–2012 as five-year moving averages (grey line). Black circles indicate the interannual variations of experimental means across all the cultivars of Luke Official Variety Trials. The figure within the panel indicates the mean genetic change in quality traits.

Interannual variation in single grain weight and grain protein concentration was high in Luke’s Official Variety Trials (Fig 5). Nonetheless, single grain weight has improved in winter wheat by 0.09 mg ya^-1^ and in winter rye by 0.18 mg ya^-1^, while grain protein concentration has declined by 0.02 and 0.05 percent unit ya^-1^, respectively. The genetic improvement in single seed weight was not apparent for turnip rape while in oilseed rape it increased by 0.02 mg ya^-1^ (Fig 6). However, seed protein concentration has declined by 0.03 in turnip rape and 0.06 percent unit ya^-1^ in oilseed rape. This coincided with significant genetic improvement in seed oil concentration: averaging 0.07 and 0.12 percent unit ya^-1^, respectively. Seed protein concentration of pea increased by 0.04 percent unit ya^-1^ and single seed weight by even 0.56 mg ya^-1^.

### Role of minor crops in crop rotations

Comparison of land use during the early CAP-period of 1995–1999 to the late CAP-period of 2007–2011 showed that spring cereal domination in field use has strengthened (S1 Table). One quarter of the fields in our five-year follow-up had only spring cereals in their crop rotation, while in 20% of fields a break-crop was used only once in the rotation. About 10% of fields had spring cereal once during their five-year rotation.

Winter wheat, turnip rape and oilseed rape were introduced more frequently to the rotation when comparing early and late CAP-periods (S1 Table). About 6% of fields had winter wheat once and 2% twice during the five-year rotation, while for rye the figures were 4% and 1%, respectively. Again 21% of fields had turnip rape once in rotation and 3% twice, while the figures for oilseed rape were 5% and 0.3%, respectively. Potato and pea were very scarcely seen in rotations. Perennial grasslands had a quite equal share of fields (4–5%) for all cases, from one to five times appearance in crop rotations, while in about 77% of field grass crops were not cultivated at all.

### Responsiveness of minor and major crops to variable conditions

**Fig 7:**
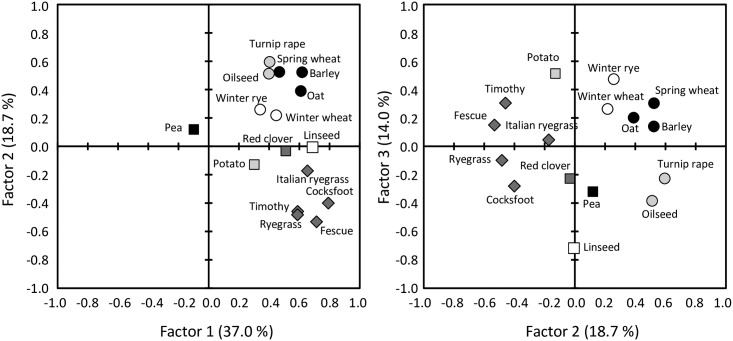
Factors 1, 2 and 3 accounting for some 70% of variation in yield and showing the differences in response of different major and minor crops to factors causing yield variability. The higher the distance between crops, the higher the difference in their response to factors causing yield variation. Data from Luke Official Variety Trials.

Three factors were found to be significant by eigenvalues and a scree plot. The analysis was used to reveal underlying response diversity: crops were grouped according to their differences and/or similarities in response to experienced weather conditions like temperature and precipitation causing variation in yields. This assessment indicated some differences in grouping of major and minor crops (Fig 7). Factor 1, with the highest loadings, indicated that especially pea differed markedly from any other crop. According to factor 2, response of major spring cereals was close to that of rapeseed, which again markedly differed from the response of grass crops, potato and pea and also from winter cereals to some extent and some other minor crops. Factor 3 further highlighted differences in responses of rapeseed and spring cereals.

## Discussion

### Declining role of minor crops in land use

Diverse cropping systems were the means to maintain productivity in agricultural systems since antiquity until their breakdown in many regions in the last century [27]. The role of major and minor crops in crop rotations has shifted markedly since the 1920s in Finland, when the total cultivated land area has ranged from 2.1 to 2.8 million hectares, depending on field area left as fallow. The main drivers for the past land use changes were technological and structural changes in agriculture, changes in trade, policy, markets and prices as well as general increase in food security. The turning point for the current two groups of major crops was 1970, when area under cereals permanently exceeded that for grassland (S3 Fig). Area allocated to hay and pasture has declined, whilst that for silage increased, and spring barley became the most common cereal by the early 1970s when the area under oats declined as tractors replaced workhorses. In the 1990s, spring wheat areas gained ca. 200 000 ha (S1 Fig). Potato and winter rye were important staple crops in the early 20^th^ century, but today rye corresponds only to 10% and potato 30% of the cropping area in 1920–1940 representing a switch from their major crop roles to minor crops. Winter wheat and pea have always been minor crops, whereas production areas for rapeseed have gradually increased and reached 80 000 ha (in 2010 even reaching 160 ^000 ha, following the very low cereals prices of 2009) since the introduction of high-quality spring turnip rape cultivars in the early 1970s. Rapeseed has approached the role of a major crop, but only temporarily, as the area declined again since 2012 to only 40 000–50 000 ha due to a reduced demand for oilseeds in the food industry, increased cereals prices, and serious uncertainties in yields and crop protection risks [28,29].

Switching of some major crop roles to minor crops and shifts in cultivation areas of major crops at the expense of minor crops, as described above, emphasize the likely risks for loss of diversity in crop rotations. This was also apparent when the early CAP-period of 1995–1999 was compared to the late CAP-period of 2007–2011: spring cereal domination in land use was even further strengthened (S1 Table)—despite the fact that this case study region was one of the most favourable areas for special crop production in Finland. During 2007–2011 even 28% of fields had spring cereals as the sole crops in their crop rotations and in 20% of fields that were dominated by spring cereals had a break-crop once in a five-year rotation. Again only 10% of fields had spring cereal only once within the five-year rotation. Regarding the role of minor crops in crop rotations, winter wheat, turnip rape and oilseed rape were introduced more frequently to the rotations during the late CAP-period (S1 Table). Both winter cereals appeared only one or two times in a five-year rotation with the shares of 6% and 2% of fields allocated for wheat and 4% and 1% for rye, respectively. Break-crops differ in their value for the successive crop [30] and there are limitations on how frequently some minor crops can be break-crops in a rotation to avoid elevation in crop protection risks. Farmers have followed these guidelines carefully with rapeseed and hence, lack of proper time between rapeseed in rotations is not the driver for expanded yield gaps in rapeseed during EU membership (Fig 4). The data for faba bean was too scarce to assess any yield trends, but faba bean has been an emerging minor crop exceeding potato and equalling pea with its 2% share of fields.

Grass crops are considered to be major crops but this only applies to the northern part of the country. Due to strong regional fragmentation of dairy and crop production in Finland, driven by the massive structural changes that agriculture went through since the 1970s, grasslands are rare in the southern part of the country: 77% of the fields lack any perennial grasslands in their five-year rotation. These southern regions have potential for good quality hay production for horses. However, such demand is limited and often local. There was also some seed production of grass crops in the southwestern Finland until the adoption of EU regulations. Thereby, southwestern Finland has increasingly specialized in cereals production and pig and poultry husbandry. However, some AEP measures have been introduced to maintain some perennial grassland in southern Finland. There are typically non-productive grasslands like nature managed fields mainly located in neglected, low-productive fields. As such, they do not diversify crop rotations and cereal monocultures.

Different approaches used to study land allocation to minor and major crops underline the role of sufficiently stable local demand and sufficiently high prices as a pre-requisite for significant expansion of minor crops [18]. Since the prices and yields of minor crops are also relatively volatile between years, there is also a need for effective policy instruments to encourage farmers to keep minor crops in their crop rotations despite occasional low profitability. Greening measures in the CAP 2015–2020 require avoiding monoculture cropping systems. As a sole measure, however, greening requirements are not likely to be sufficient for any significant increase of minor crop areas. This is because there is still limited (local) demand for the minor crops, while greening obligations can also be realised in other ways, e.g. by adopting AEP measures, or allocating land for set aside. CAP farm payments were decoupled from production since 2006 and relatively low hectare-based support payments (e.g. some 50 € ha^-1^) for minor crops (with budgetary limits) cannot be their main drivers of promotion. Since market demand should drive production and land use in the current CAP, it seems that market demand and prices are not sufficient to push through diversification actions.

Efficiency of diversification to mitigate weather variability is dependent on a magnitude of differences in crop responses [31]. Diversification of crop rotations with the current minor crops has potential to increase resilience as our long-term datasets showed differences in yield responsiveness to be particularly high between crop groups: spring cereals, rapeseed, grain legumes, potato, and grass crops. However, response diversity within each crop group was only marginal, indicating a limited capacity to improve resilience, e.g. by having different spring cereals in rotation compared to including other crop groups (Fig 7). These findings support the concern that EU greening payments, accepting allocation of land to, e.g. three small grain cereals, does not truly increase biodiversity and is not apt to improve resilience to weather variability.

### Yields, yield gaps and quality changes in minor crops over time

When compared to spring wheat as a major crop with expanding land area in Finland, all minor crops have undergone a lowering rate of national yield improvements when three time periods were compared (Figs 2 and 3). National yields of major spring cereals have stagnated since Finland joined EU and CAP [1], while in rapeseed, potato and grass crops the yields have declined. In winter cereals and pea, yields have levelled-off or if increased have done so only marginally (Table 1). Laidig et al. [32] reported genetic yield improvements, without marked agronomic progress, for a high number of field crops (except for fodder grasses) in Germany, as did Mackay et al. [33] for cereals and oilseed rape in the UK for the last couple of decades. This agrees with our study, as plant breeding has been successful in all minor crops (as well as for major crops) in Finland, and often most in the most recent period, but yield gaps between potential and actual farm yields have increased, except for winter wheat. This alarming finding of increasing yield gaps was most evident for turnip rape and potato, meaning that their national yields have declined, or increased less compared to increases in genetic yield potentials depending on time period. Our method, which considered comprehensively regional limitations, indicated the current yield gap to be about 1000 kg ha^-1^ for winter wheat, contrary to the much greater gap of 2500 kg ha^-1^ for winter rye.

This study suggests that in some cases increases in genetic yield potential have been associated with declines in quality traits. Interannual variation in single grain weight and grain protein concentration was high (Fig 5), but there was a clear trend of increased grain size in winter cereals with a concomitant slight decline in grain protein concentration. This was also apparent for oilseed rape, but for turnip rape only regarding a decline in seed protein concentration (Fig 6). These changes in rapeseed, however, coincided with significant genetic improvements in seed oil concentration, which again indicates trade-offs between these quality traits in breeding programs. Contrary to other crops, seed protein concentration of pea increased as did single seed weight. For potato, genetic improvements in the share of commercially acceptable quality and sensitivity of darkening as raw or boiled were evident (S4 Fig). These findings indicate that plant breeding has not only been successful in shifting genetic yield potentials of minor crops but also their quality, although some typical trade-offs were apparent [see e.g. [34,35]]. Hence, the influences of privatising plant breeding and focussing breeding on major crops at the expense of minor crops [14] has not yet caused a lack of genetic improvement in minor crops, although the pace of improvements has been lower compared to the emerging major crop, spring wheat. As we lack large-scale farm surveys of yield and quality that were available for major spring cereals [1], the changes in quality of farmer’s yield cannot be comprehensively characterized for minor crops. The only exception is winter rye, for which genetic drawbacks in grain protein concentration have coincided with the reduced use of N input, which together likely contribute to reductions in on-farm grain protein concentrations established for the AEP period (data not shown).

### Drivers for changes in yield trends

The yield stagnation in major spring cereals was attributable to many contemporary changes in agricultural practices, driven by changes in prices, agricultural policies and subsidies: e.g., reduced use of N fertilizers, increasing share of leased land, neglected basic investments (e.g. on liming and sub-soil drainage), introduction of direct-drilling and minimum tillage, insufficient use of fungicides, soil compaction and expanded area under organic production, to mention only some major changes in crop management and cropping systems [36–39]. All these drivers contribute differently to the stagnation of national major cereal yields [1]. These examples highlight the need for R&D investments and concrete actions to facilitate the dramatic, contemporary changes in cropping systems. It is, however, obvious that the investment in minor crops to better adapt them to changing production systems has been less intensive than that for major crops. On the other hand, for spring cereals, reduced N application rates were concluded to be among the main factors contributing to the inability of the current farming systems to exploit the improved genetic yield potential of modern cultivars [1]. This is not, however, likely with the minor crops such as N-fixing grain legumes, crops like potato with reasonably intensive production and rapeseed. Rapeseed has undergone yield decline without being primarily driven by reduced fertilizer use: it has the lowest yield removed N, and thereby, seemingly low N use efficiency [40], which again may be partly attributable to compacted soils impeding the root penetration of rapeseed [41]. On the other hand, turnip rape has faced major challenges during the 2000s caused by insufficient and even reduced availability and use of chemical control agents while plant protection risks have become more severe [29]. The most dramatic, possibly even final drawback for rapeseed cultivation was when EU banned the use of neonicotinoids (Commission Implementing Regulation (EU) No 485/2013) without alternative insecticides or control means. This contributes to the current reluctance of farmers to continue cultivating rapeseed in Finland. Due to the lack of any markets for silage, production areas for silage and hay in each farm are planned to be sufficient in the most challenging, dry years. Hence, the yield potential is not necessarily utilized in high-yielding conditions. This likely explains stagnant grass crop yields (Fig 3), in spite of genetic gains in their potential yields.

Minor crops are considered to require more farmer’s attention and additional inputs like pesticides compared to the major cereals, but their yields vary more with even a risk for total crop failures [22]. The trend of increase in average farm size since Finland joint EU may have hampered farmer’s capacity to monitor and also carry out the management practices in a timely manner, which is especially harmful for special crops needing e.g. timely and repeated pest control. It is shown, using economic farm level models as well, that decreasing crop prices or increased input prices imply reduced use of inputs such as liming, fungicides and fertilisers, reduces the number crops in rotation, and leads to monoculture. Risk aversion behaviour of farmers plays a role also in Finland where yield variability is relatively high [17,18]. Increased input prices since 2000 and increasing volatility of prices and gross margins since 2006 have favoured simple rotations, reduced use of inputs and discouraged diverse rotations. Decoupled CAP area payments provide a possibility for cost minimisation at farms with relatively high production costs.

Unfortunately, cost minimization does not match well with the additional investment and input needs that are typical for special crop production. Farmer risk-aversion may hence further increase the yield gaps and limit their closure [42]. However, comparison of share of yields having acceptable quality (S2 Fig) indicated that even though the regional differences were high for some crops compared to spring wheat, by allocating more land to minor crops in areas with only reasonable yield variation, is a means to improve yield stability of minor crops and increase resilience of cropping systems as a whole [12,28]. Diverse cropping systems, especially when compared to cereal monocultures, have also potential to mitigate nutrient loads that are increased at high latitudes by climate change [43]. In addition to maintaining the breeding programs for minor crops and thereby, enhancing potential yields, quality and stability, as well as making agronomic advancements, it is essential to support expansion of minor crops in land use and crop rotations by supportive market developments and effective policy instruments in Europe [44].

## Conclusions

The AEP, an important component of CAP, has been successful in terms of reducing nutrient use and decreasing nutrient balances. However, our results with neglected minor crops question whether the environmental sustainability goals are attained only partly and in the short term, but not necessarily in the long term. This proved to be a justified concern with major crops as recent assessment indicated stagnation in yields and declines in quality traits. However, this study further deepens the concern of achieving advances in sustainability, as it indicates how the agricultural policies in the prevailing market and price environment have led to the strengthened role of only a couple major crops, neglecting large-scale diversification activities although they are of utmost importance when aiming to improve biodiversity in agriculture and rural landscapes. Therefore, optimization of agricultural land use, as a central element of sustainable intensification actions in high latitude cropping systems, is at the core of our current research activities that aim to develop agriculture not only towards better environmental sustainability, but coupling success in that with better economic profitability and social acceptability.

## Supporting Information

S1 Fig Changes in field area devoted to minor crops crops (symbol for each crop group in the right margin) since 1920 in Finland with spring wheat (in the upper panel with open circle) as a reference of major crop. Data from Luke Statistics Services [26].
(TIF)

S2 Fig Interannual variations in proportion of yield with acceptable quality in Finland in winter cereals, potato, pea and hay for 1988–2006. Spring wheat is shown as a reference of major crop. The black square is the national mean while the line indicates the spatial variation depending on year. Data from Luke Statistics Services [26].
(TIF)

S3 Fig Changes in land use of cereal crops, grassland and fallow since 1920 in Finland. Symbol indicating each crop group is shown in the right margin next to the end-tail of each trend. Data from Luke Statistics Services [26].
(TIF)

S4 Fig Genetic improvements in sensitivity of darkening as raw and boiled of winter potato in 1970–2005 as five-year moving averages (grey line). Black circles indicate the interannual variations of experimental means across all the cultivars of Luke Official Variety Trials. The figure within the panel indicates the mean genetic change in quality traits.
(TIF)

S1 Table The share of fields having none or one to five times different minor crops in their rotations within two five-years periods compared to the dominating crop, spring cereals. The data covers 70 000 fields that are located on the prime crop production region of Finland having thereby the highest potential for cultivation of minor crops. Data from Mavi.
(DOCX)
