# Supplementary figures and images for: Land Use, Yield and Quality Changes of Minor Field Crops: Is There Superseded Potential to Be Reinvented in Northern Europe?
Source: PLoS One. 2016 Nov 21;11(11):e0166403. doi: 10.1371/journal.pone.0166403 (PMC5117691; doi:10.1371/journal.pone.0166403)

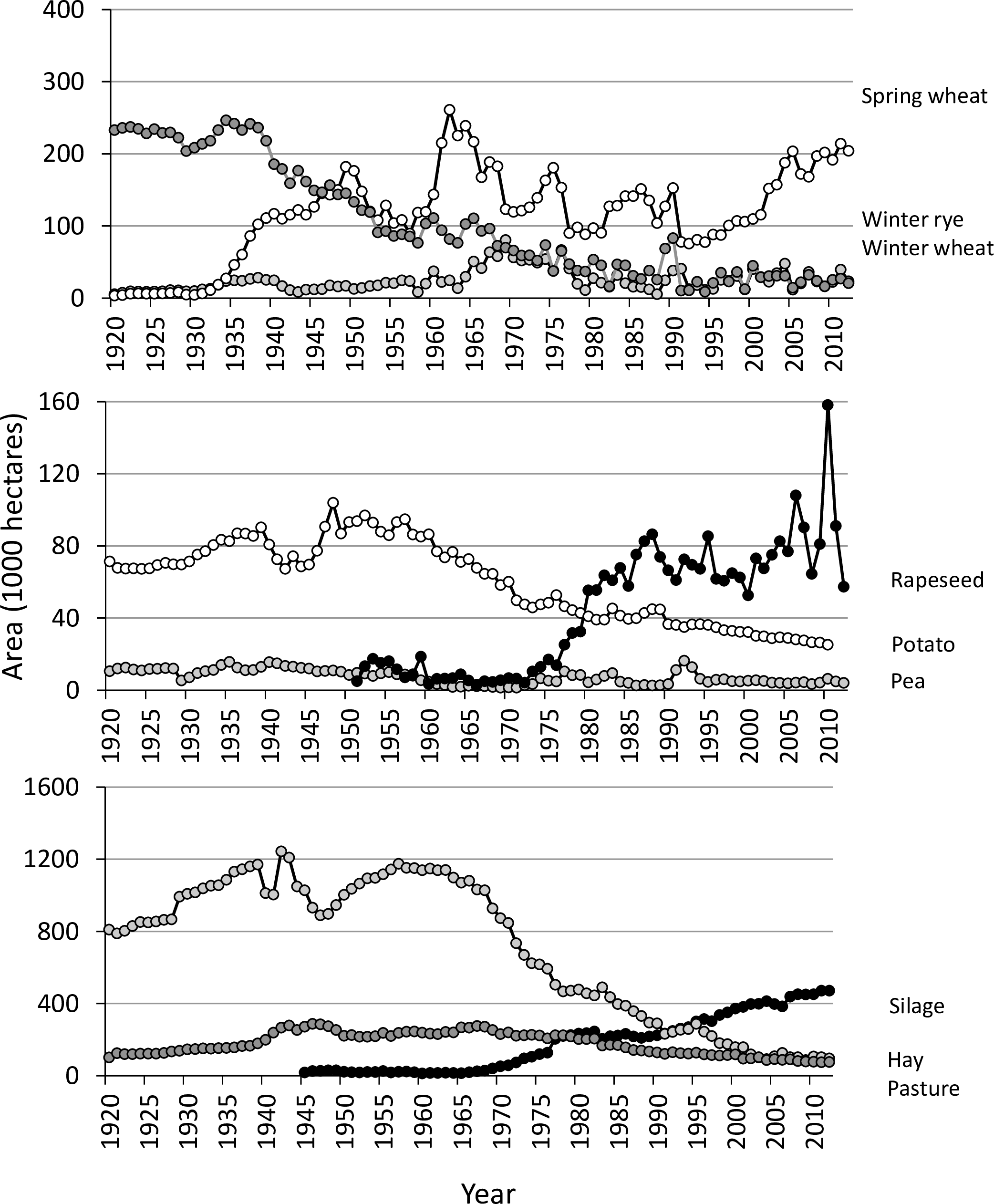

Supplement: S1 Fig — Data from Luke Statistics Services [26]. (TIF) [file pone.0166403.s001.tif]

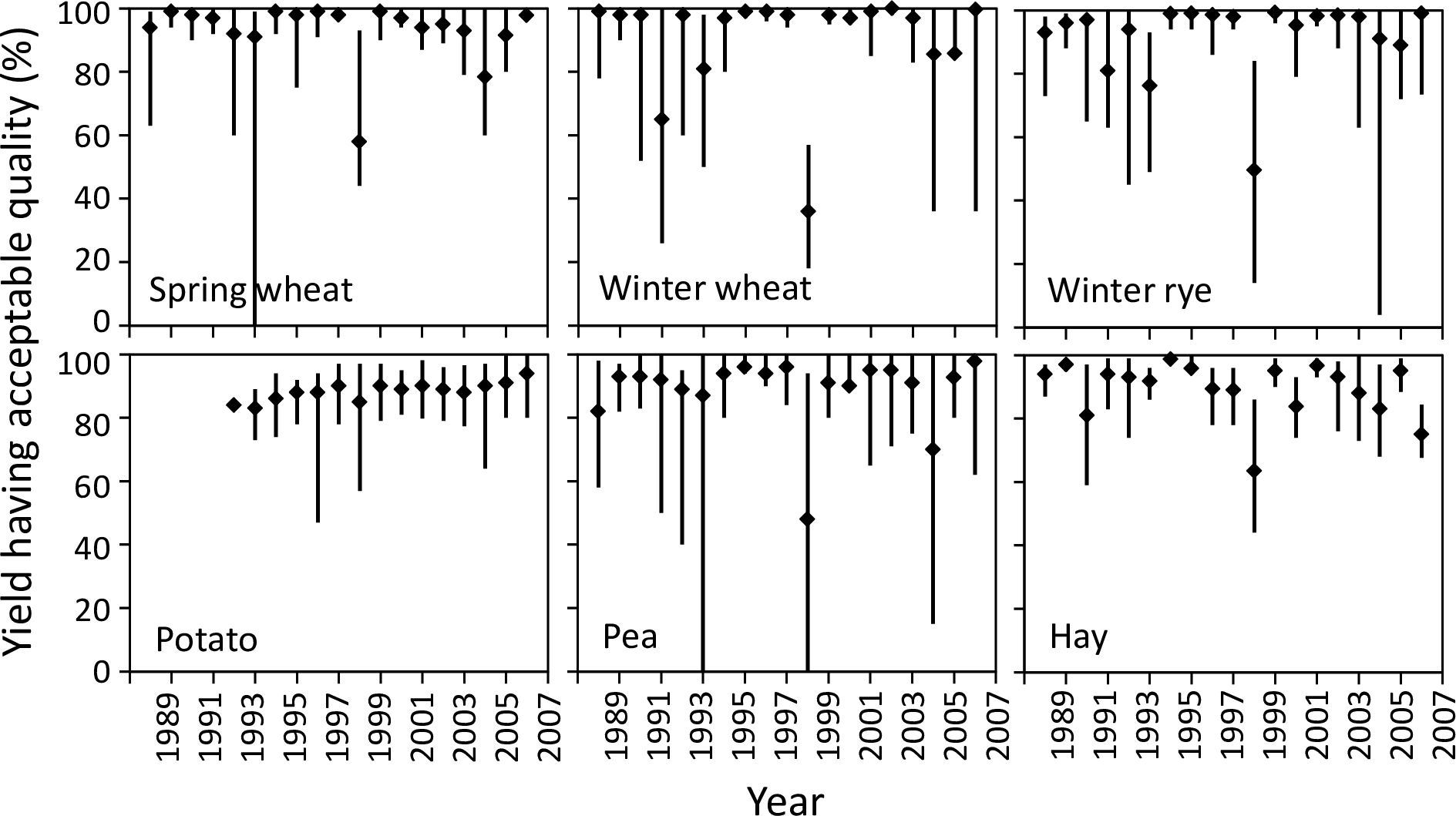

Supplement: S2 Fig — Spring wheat is shown as a reference of major crop. The black square is the national mean while the line indicates the spatial variation depending on year. Data from Luke Statistics Services [26]. (TIF) [file pone.0166403.s002.tif]

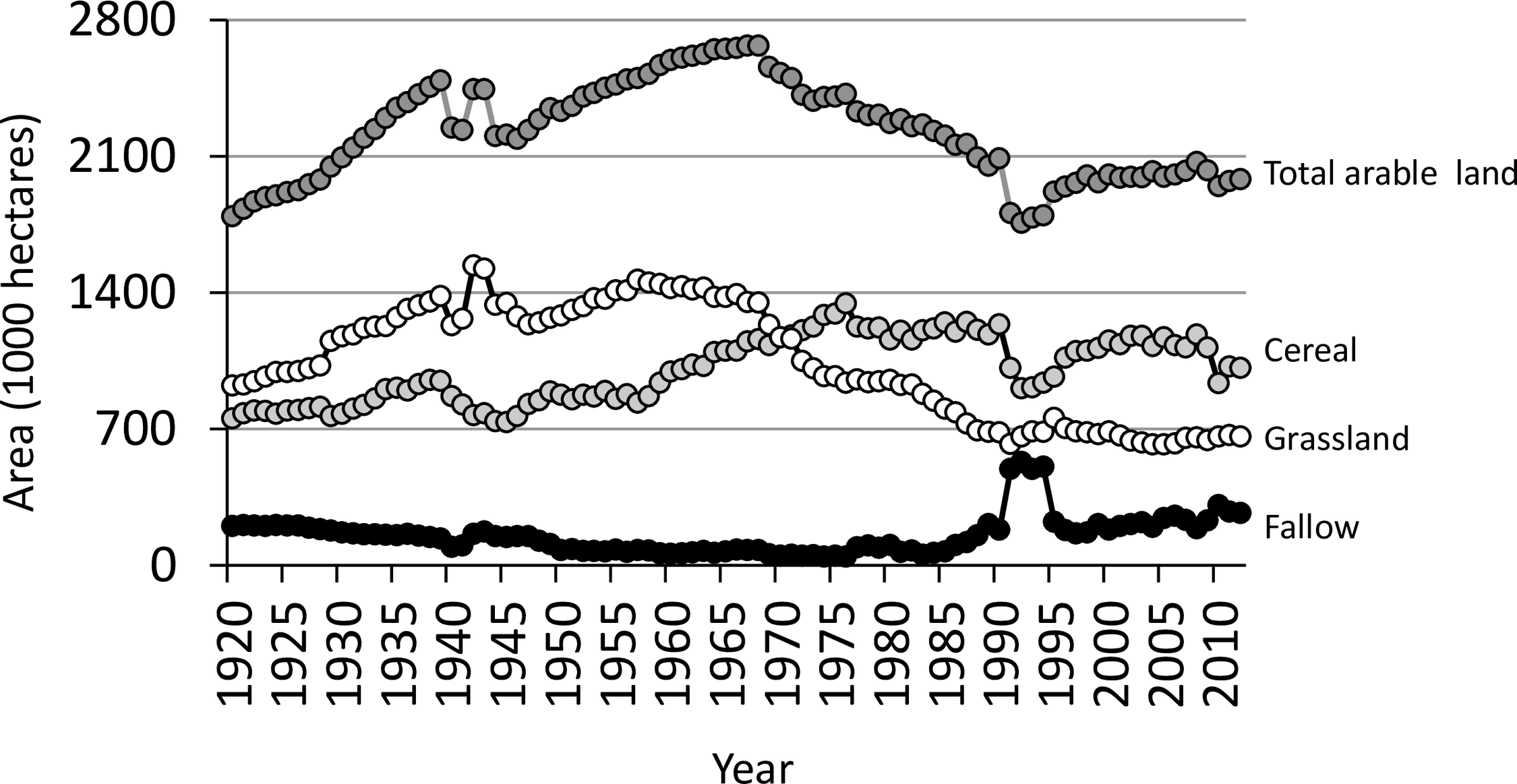

Supplement: S3 Fig — Symbol indicating each crop group is shown in the right margin next to the end-tail of each trend. Data from Luke Statistics Services [26]. (TIF) [file pone.0166403.s003.tif]

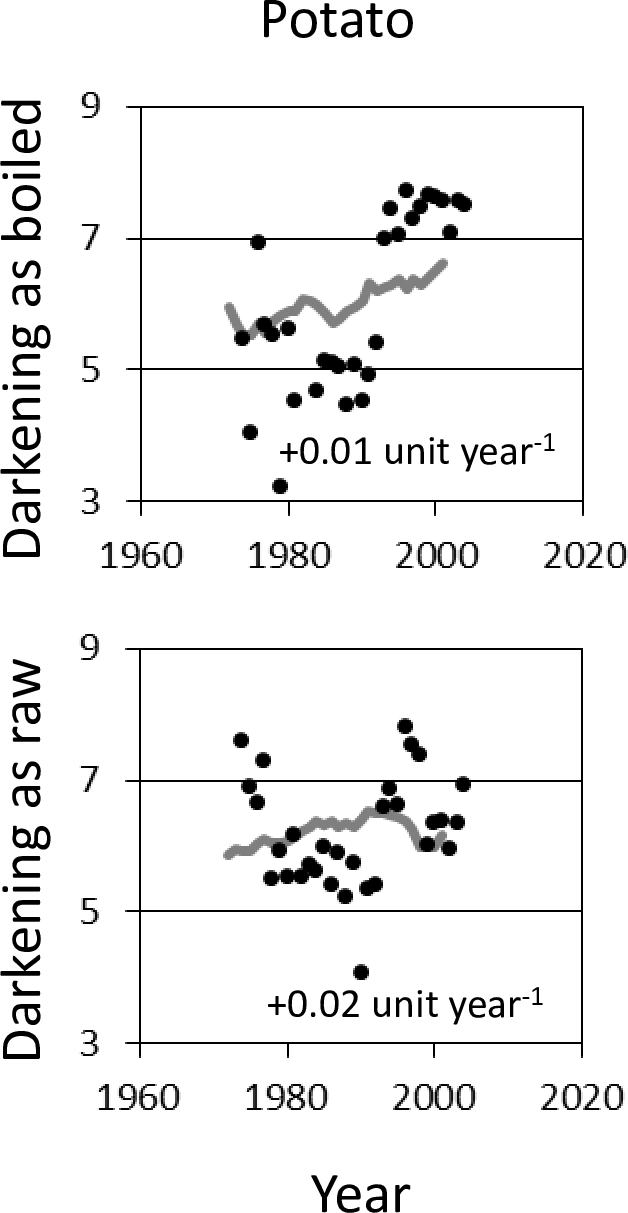

Supplement: S4 Fig — Black circles indicate the interannual variations of experimental means across all the cultivars of Luke Official Variety Trials. The figure within the panel indicates the mean genetic change in quality traits. (TIF) [file pone.0166403.s004.tif]
