# Supplementary material for: Land Use, Yield and Quality Changes of Minor Field Crops: Is There Superseded Potential to Be Reinvented in Northern Europe?
Source: PLoS One. 2016 Nov 21;11(11):e0166403. doi: 10.1371/journal.pone.0166403 (PMC5117691; doi:10.1371/journal.pone.0166403)
Supplement: S1 Table — The data covers 70 000 fields that are located on the prime crop production region of Finland having thereby the highest potential for cultivation of minor crops. Data from Mavi. (DOCX) [file pone.0166403.s005.docx]

S3 Table. The share of fields having none or one to five times different minor crops in their rotations within two five-years periods compared to the dominating crop, spring cereals. The data covers 70 000 fields that are located on the prime crop production region of Finland having thereby the highest potential for cultivation of minor crops. Data from Mavi.

| No. of cases with a crop in rotation | Proportion of a crop in rotation | | | | | | | |
| --- | --- | --- | --- | --- | --- | --- | --- | --- |
|  | Spring cereals | Winter wheat | Winter rye | Turnip rape | Oilseed rape | Potato | Pea | Perennial grasslands |
|  |  |  |  |  |  |  |  |  |
| 1995‒1999: |  |  |  |  |  |  |  |  |
| None | 18.4 | 93.6 | 92.7 | 79.5 | 99.4 | 99.0 | 98.1 | 74.5 |
| 1 | 8.7 | 3.8 | 5.7 | 16.6 | 0.6 | 0.3 | 1.7 | 5.3 |
| 2 | 11.2 | 1.8 | 1.4 | 3.6 | 0.0 | 0.2 | 0.2 | 4.8 |
| 3 | 15.7 | 0.6 | 0.2 | 0.3 | 0.0 | 0.2 | 0.0 | 5.6 |
| 4 | 21.8 | 0.2 | 0.0 | 0.0 | 0.0 | 0.1 | 0.0 | 4.9 |
| 5 | 24.2 | 0.0 | 0.0 | 0.0 | 0.0 | 0.2 | 0.0 | 4.9 |
|  |  |  |  |  |  |  |  |  |
|  |  |  |  |  |  |  |  |  |
| 2007‒2011: |  |  |  |  |  |  |  |  |
| None | 16.2 | 91.5 | 95.0 | 76.0 | 94.5 | 99.3 | 98.3 | 76.6 |
| 1 | 10.3 | 5.9 | 4.0 | 21.2 | 5.2 | 0.2 | 1.6 | 4.3 |
| 2 | 12.1 | 2.0 | 0.9 | 2.7 | 0.3 | 0.2 | 0.1 | 4.2 |
| 3 | 14.3 | 0.5 | 0.1 | 0.1 | 0.0 | 0.1 | 0.0 | 4.5 |
| 4 | 19.4 | 0.1 | 0.0 | 0.0 | 0.0 | 0.1 | 0.0 | 4.7 |
| 5 | 27.7 | 0.0 | 0.0 | 0.0 | 0.0 | 0.1 | 0.0 | 5.7 |
|  |  |  |  |  |  |  |  |  |
